# An unsuspected histopathological finding —concomitant IgA nephropathy in a patient with ANCA-associated vasculitis: a case report and literature review

**DOI:** 10.3389/fimmu.2023.1227878

**Published:** 2023-08-15

**Authors:** Maciej Tota, Piotr Donizy, Martyna Byrska, Magdalena Krajewska, Mariusz Kusztal

**Affiliations:** ^1^ Faculty of Medicine, Wroclaw Medical University, Wrocław, Poland; ^2^ Department of Clinical and Experimental Pathology, Wroclaw Medical University, Wrocław, Poland; ^3^ Department of Nephrology and Transplantation Medicine, Wroclaw Medical University, Wrocław, Poland

**Keywords:** IgA nephropathy, ANCA-associated vasculitis, granulomatosis with polyangiitis, GPA, PR-3 ANCA, kidney biopsy, treatment, CRP

## Abstract

Although associations of IgA nephropathy (IgAN) and ANCA-associated vasculitis (AAV) have been described, this coexistence scarcely occurs and requires multidisciplinary management. Herein, we discuss a course of treatment introduced in a patient with two exacerbations. Furthermore, alterations in histopathological images between two kidney biopsies are presented. The applicability of traditional inflammatory markers, e.g., CRP, in monitoring disease severity in AAV and IgAN is limited. Based on our patient and current literature, we suggest ANCA testing in patients with rapidly progressing IgAN for therapeutic and prognostic purposes. As regards the therapy of IgAN associated with AAV, aggressive immunosuppressive regimens with methylprednisolone and cyclophosphamide are recommended. Alternatively, methylprednisolone with rituximab, plasma exchange, mycophenolate mofetil, and intravenous immunoglobulin (IVIG) could also be considered.

## 1 Introduction

IgA nephropathy (IgAN) is the most common glomerulonephritis worldwide (1). Numerous disease entities, such as liver, dermatological, gastrointestinal, autoimmune, oncological, and respiratory disorders, as well as infectious, iatrogenic (drugs), and environmental factors, are recognized as triggers for IgAN development (2). As regards the pathophysiology of IgAN, the four-hit hypothesis is suggested. Elevated levels of galactose-deficient IgA1 (hit 1.) and the production of specific anti-glycan antibodies (hit 2.) cause the formation of pathogenic IgA1-containing immune complexes (hit 3.). The subsequent accumulation causes the release of cytokines and extracellular matrix proteins (hit 4.), contributing to renal dysfunction (3).

Anti-neutrophil cytoplasmic antibody (ANCA)-associated vasculitis (AAV) is a condition that causes severe systemic small-vessel vasculitis and is defined by the emergence of autoantibodies against the neutrophil proteins leukocyte proteinase 3 (PR3-ANCA) or myeloperoxidase (MPO-ANCA) (4). Three major ANCA-associated vasculitides have been identified according to the 2012 Chapel Hill Consensus: granulomatosis with polyangiitis (GPA), microscopic polyangiitis (MPA), and eosinophilic granulomatosis with polyangiitis (EGPA) (5). More than 75% of patients with AAV have renal involvement, characterized by rapidly progressing glomerulonephritis. Untreated AAVs have a less than one-year survival rate. The 5-year kidney survival rate is 70–75% in patients receiving the proper immunosuppressive medication (6). Genetics, environmental factors, and reactions of the innate and adaptive immune systems all play a role in the complex origin and pathophysiology of AAV (7).

Two studies on the prevalence of AAV in IgAN showed similar results: 1.2% (4/330) and 1.5% (35/2,390) of IgAN biopsy-proven patients were ANCA-positive (8, 9). Hence, the coincidence of AAV and IgAN is rare and few reports have described it. The aim of our study was to summarize the current literature on the suggested common pathomechanism, clinical presentation, and management of AAV and IgAN. We also present a successful course of treatment in a patient with AAV and IgAN and discuss the role of traditional inflammatory markers in monitoring disease activity.

## 2 Case report

In January 2019, a 56-year-old caucasian man was referred to the nephrology department due to fatigue, joint and muscle pain, arterial hypertension, hemoptysis, and bloody discharge from the nose lasting for two months. Chest computed tomography (CT) performed in an outpatient setting revealed diffuse alveolar hemorrhage (DAH) with a 20% alteration of lung parenchyma. Furthermore, abdominal ultrasonography (USG) showed mild dilatation of the pelvicalyceal system in the left kidney. The family history was positive for cerebral stroke and negative for autoimmune diseases.

**Figure 1:**
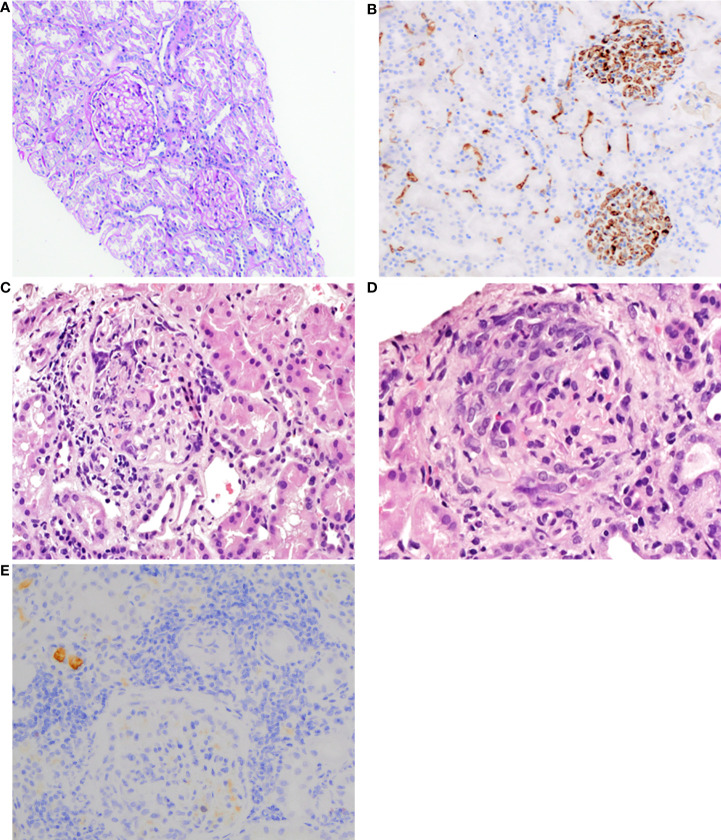
**(A) **First kidney biopsy. PAS staining of the cortex shows normal to mild mesangial hypercellularity with normal tubules and interstitium (100×). **(B) **First kidney biopsy. Immunohistochemistry for IgA reveals intense granular staining, predominantly mesangial (200×). **(C) **Second kidney biopsy. Glomerulus with fibrinoid necrosis and karyorrhexis. HE staining (400×). **(D)** Second kidney biopsy. Glomerulus with a cellular crescent. HE staining (600×). **(E)** Second kidney biopsy. Immunohistochemistry reveals a significant reduction of IgA reactivity in glomeruli (200×).

**Figure 2:**
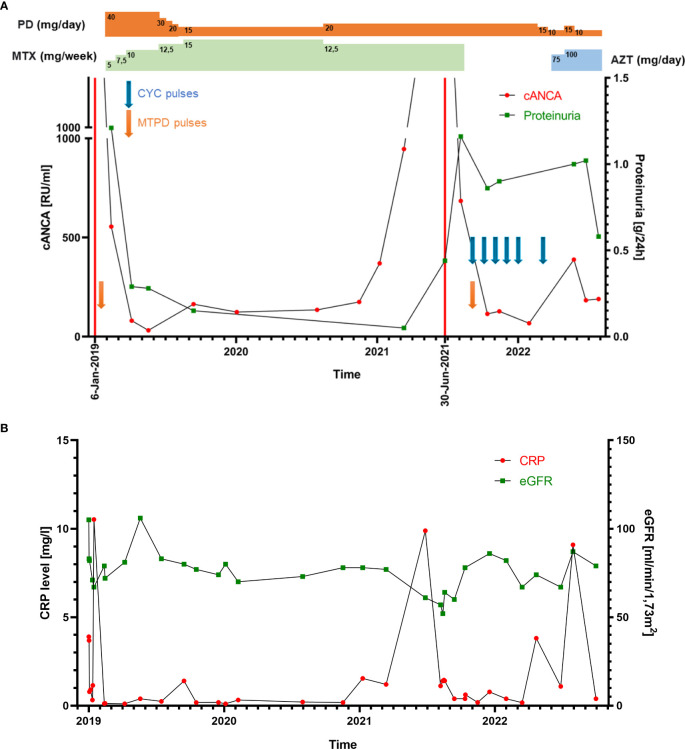
**(A) **Line graph showing the patient’s cANCA (PR3-ANCA) levels and proteinuria throughout therapy. The two exacerbations are marked with red lines (6 January 2019 and 30 June 2021), consistent with cANCA and proteinuria level elevation. PD, prednisone; MTX, methotrexate; AZT, azathioprine; CYC, cyclophosphamide; MTPD, methylprednisolone. **(B) **Line graph representing the patient’s CRP level and eGFR ratio changes.

On admission, petechiae on the legs were observed. Laboratory studies revealed positive PR3-ANCA (>1000 RU/ml) and ANA (1:320), proteinuria (0.33–0.44 g/24 h), and microhematuria. MPO-ANCA and anti-GBM antibodies were negative. Moreover, mild inflammation (C-reactive protein (CRP): 11 mg/L; erythrocyte sedimentation rate (ESR) within the normal limit: 19 mm/h) and normal kidney function (estimated glomerular filtration rate (eGFR): 108 ml/min/1.73 m^2^). During the first hospitalization, 40 mg of methylprednisolone (MTPD) was introduced, resulting in a nearly complete resolution of the reported symptoms. One month later, hematuria persisted and PR3-ANCA levels were 555 RU/ml. Surprisingly, a kidney biopsy showed none of the typical morphological features of ANCA-related vasculitis but IgA nephropathy with an Oxford score MEST-C: M_1_, E_0_, S_0_, T_0_, C_0_ (**Figures 1A, B**). Such a possible negativation of proliferative lesions could have been a result of commenced immunosuppressive treatment. Therefore, MTPD pulses 4x250 mg with conversion to prednisone 40 mg/day and methotrexate (MTX) 7.5 mg were introduced. At the follow-up visit in April, the second CT demonstrated a complete resolution of previous changes. All symptoms were completely resolved. The MTX dose was increased to 10 mg/week and MTPD was tapered to 12 mg/day. Although laboratory tests and the patient’s clinical state were well controlled, a gradual increase in PR3-ANCA antibodies was noted (**Figure 2A**). CRP levels were within normal limits (**Figure 2B**).

In May 2021, the second exacerbation occurred. The patient presented at the ophthalmologist’s office due to redness and eye pain. Episcleritis was diagnosed and treated with loteprednol and brimonidine/timolol eye drops. In the next month, the patient developed muscle and joint pain that resolved spontaneously after a few weeks. Due to the overall clinical picture, a second kidney biopsy was performed. It revealed ANCA-associated glomerulonephritis, namely endocapillary proliferation within 8/20 of the glomeruli, with segmental fibrinoid necrosis, karyorrhexis, and prominent cellular crescents in three glomeruli. A low intensity of chronic inflammation (TIN Grade 0/1) in the interstitium with mild fibrosis was observed (2–3% of the available renal cortex) (**Figures 1C, D**). An immunohistochemistry examination of the kidney specimen did not show IgA deposition (**Figure 1E**). Chest CT scans showed irregular linear opacity with traction of the adjacent lung parenchyma seen in the right upper lobe with a size of 2.3x2.0 cm; signs corresponding to inflammation. Otherwise, normal lung parenchyma. The patient received 1 g of cyclophosphamide (CYC), and MTPD pulses 2x500 mg with conversion to prednisone 35 mg/day. In subsequent monthly follow-up visits, the patient received 800 mg of CYC (a total dose of 5000 mg; 62.5 mg/kg) (**Figure 2A**).

In March 2022, azathioprine (AZT) was introduced (75 mg, which was increased to 100 mg after four weeks). In October 2022, remission was achieved based on clinical and laboratory images, with a Birmingham Vasculitis Activity Score (BVAS) = 1.

## 3 Discussion

In recent years, MTX versus CYC for induction of AAV remission has been a matter of debate. Considering CYC adverse events, MTX was suggested in patients with non-life-threatening AAV (10). Hence, our patient was administered with MTX. However, recently published studies have shown that MTX use is associated with a higher relapse rate and late accrual damage compared to CYC (11). Thus, CYC with glucocorticoids over MTX is recommended for induction of remission of AAV according to KDIGO 2021 Guidelines. Alternatively, rituximab with glucocorticoids may be considered. In life-threatening cases, plasmapheresis is recommended. Conversely, in a no-organ-threatening course, mycophenolate mofetil may be administered (11).

Our report represents a patient with AAV and a first histopathological diagnosis unequivocal for IgAN. Traditional inflammatory marker CRP ratios in our patient were significantly elevated only during exacerbations. Thus, we conclude that CRP has a limited role in assessing disease activity. Our thesis is consistent with previous reports demonstrating weak or no correlation between CRP and BVAS (12, 13). Similarly, other inflammatory markers, e.g., ESR, procalcitonin, and calprotectin, were not applicable in monitoring patients with AAV (14, 15).

**Figure 3:**
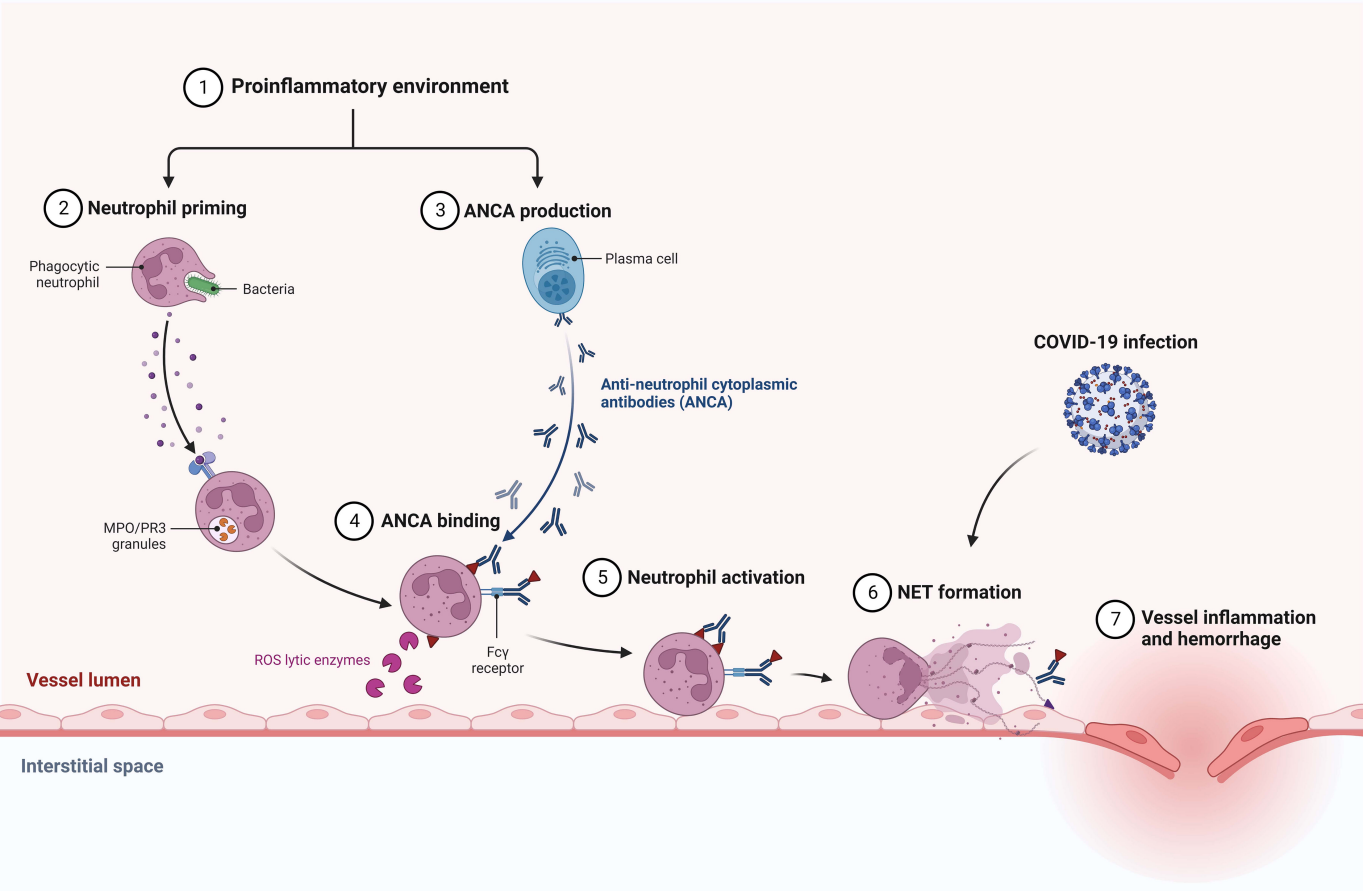
Suggested association between COVID-19 infection and AAV development via neutrophil extracellular traps (NETs) formation.

Ozcan et al. described the coexistence of primary IgAN diagnosed two years prior and a novel AAV diagnosis that may had been provoked by COVID-19 infection (**Table 1**). Similar to our patient, a CT scan showed an alveolar hemorrhage (16). The link between COVID-19 and AAV and IgAN has not been elucidated yet. Comparably, nonspecific upper respiratory tract infection preceded AAV and IgAN diagnosis in our patient. Growing evidence suggests that the formation of neutrophil extracellular traps (NETs) during COVID-19 infection may lead to AAV development (31, 32) (**Figure 3**). As concerns kidney diseases, lupus nephritis, anti-glomerular basement membrane disease, thrombotic microangiopathies, and diabetic nephritis are also suggested to share the contribution of NETs in pathogenesis (33). Such a possible link was not corroborated for IgAN and should be evaluated by future studies.

**Table 1:** Summary of to-date published case reports on AAV and IgAN coexistence.

Patient characteristics	Serology	Symptoms	Histopathological image	Introduced treatment	Response to treatment	Year of publication	Reference
26 y.o., male	anti-MPO positive, anti-PR3 negative	severe progressive shortness of breath, hemoptysis, hematuria, dry cough, weakness, loss of appetite	mesangial proliferation, with no crescents and no findings related to necrotizing vasculitis; Oxford score MEST: M_1_, E_1_, S_1_, T_0_	1 g/day intravenous (IV) methylprednisolone (MTPD) for 3 days → prednisolone 1 mg/kg/day + 1 g/month cyclophosphamide (CYC)	improvement	2022	(16)
66 y.o., male	anti-MPO positive, anti-PR3 negative	10-month history of weakness, weight loss, arterial hypertension	segmental endocapillary hypercellularity, synechiae in the Bowman’s capsule, fibrous crescents 2 of 7 glomeruli, diffuse interstitial fibrosis and tubular atrophy (25–30%); focal proliferative IgAN; Oxford score MEST-C: M_1_, E_1_, S_1_, T_1_, C_2_	500 mg/day IV MTPD for 3 days and 32 mg daily orally and IV CYC (800 mg every 4 weeks), maintenance therapy with mycophenolate mofetil (MMF) 2 g/day	remission	2020	(17)
12 y.o., female	anti-MPO positive	macroscopic hematuria, joint pain, fever, anemia	mesangioproliferative glomerulonephritis with cellular and fibrocellular crescents (21%), glomerular sclerosis (37%), tubular atrophy and interstitial fibrosis (10%); Oxford score MEST-C: M_1_, E_1_, S_1_, T_0_, C_1_	high-dose corticosteroids and CYC (cumulative dose of 3.8 g); relapse of AAV and treatment with MMF 1.480 mg daily had no effect on proteinuria → 500 mg IV rituximab (RTX) weekly × 4	improvement: RTX 1000 mg every 6 months to maintain remission		
26 y.o., male	anti-MPO positive, anti-PR3 positive	lymphadenopathy (found on screening chest radiograph performed in the context of his father’s recent diagnosis of silicosis), glomerular hematuria (>1000 RBC/L), renal impairment (serum creatinine 250 μmol/L), intermittent night sweats for 4 weeks, dark-colored urine	mild mesangial hypercellularity and one glomerulus displaying endocapillary hypercellularity, 3 of 12 glomeruli contained crescents (1 segmental necrotizing, 2 fibrocellular)	oral CYC 150 mg and prednisolone 75 mg daily	improvement	2020	(18)
51 y.o., male	anti-MPO positive, anti-PR3 positive	impaired renal function, hematuria, proteinuria, weight loss, lethargy	cellular crescents with little or no organization in 50% of glomeruli with segmental necrosis and moderate chronic damage (40% tubular atrophy) and widespread lymphoplasmacytic infiltrate, associated with tubulitis	discontinuation of propylthiouracil (PTU), high-dose pulses of IV MTPD for 3 days, carbimazole, IV CYC, oral glucocorticoids	improvement	2020	(19)
21 y.o., female	anti-MPO positive, anti-PR3 positive	fever, macrohematuria, abdominal pain, vomiting	small cellular crescents in 2 (12%) out of 17 obtained glomeruli and focal fibrin deposition with capillary wall disruption in 4 glomeruli; most glomeruli showed mild to moderate mesangial proliferation	50 mg/day of prednisolone → 10 mg/day, 1.000 mg/day of MMF → 1.500 mg/day	improvement	2019	(20)
14 y.o., female	anti-MPO positive, anti-PR3 positive	flu-like illness, periorbital edema	mesangial matrix expansion with segmental scarring; fibrocellular crescents were present in 5 (33%) of 15 glomeruli and there was extensive focal tubular atrophy; segmental fibrinoid change was found in several glomeruli	discontinuation of PTU, 60 mg/day of prednisone, 100 mg/day of CYC, prednisone maintaining therapy	improvement	2001	(21)
46 y.o., female	anti-MPO negative, anti-PR3 positive	joint pain	Oxford score MEST-C: M_1_, E_1_, S_1_, T_x_, C_2_	CYC 125 mg/day for 6 weeks, MTPD 125 mg/day, prednisone 50–60 mg/day for 4.5 months, prednisone 80 mg every other day for 1.5 months tapered over the next 8 months	improvement	2000	(22)
45 y.o., male	anti-MPO positive, anti-PR3 positive	bilateral pulmonary infiltrates; opacified left maxillary sinus on CT scan	Oxford score MEST-C: M_1_, E_1_, S_1_, T_x_, C_2_	CYC 150–100 mg/day, prednisone 60 mg/day to 10 mg/day over 15 months	improvement		
73 y.o., male	anti-MPO negative, anti-PR3 positive	asymptomatic	Oxford score MEST-C: M_1_, E_0_, S_1_, T_x_, C_2_	CYC, prednisone	became dialysis-dependent		
12 y.o., female	anti-MPO negative, anti-PR3 positive	hemoptysis, seizures	Oxford score MEST-C: M_1_, E_1_, S_0_, T_x_, C_2_	MTPD 3 pulses, CYC 500 mg, prednisone 100 mg/every other day	improvement, became dialysis-dependent		
44 y.o., female	anti-MPO negative, anti-PR3 positive	abdominal pain, nausea, vomiting, edema, hematuria	Oxford score MEST-C: M_1_, E_1_, S_1_, T_x_, C_2_	prednisone 60 mg/day, taper over 10 months CYC 2 mg/kg/day for 3 months, relapse: prednisone 60 mg/day, CYC 2 mg/kg/day for 15 months	relapse 15 months after biopsy and 5 months after discontinuation		
68 y.o., male	anti-MPO positive, anti-PR3 negative	weakness, weight loss, hypertension	proliferative glomerulonephritis with 50% extracapillary crescents, mild focal tubular atrophy, and minimal interstitial inflammation	steroids	after a short course of steroids, renal function declined and hemodialysis was started	1997	(23)
35 y.o., male	anti-PR3 positive	lower extremity swelling, dark-colored urine, sinusitis-like symptoms (sinus congestion, postnasal drip, facial swelling, and frontal head pressure), nasal polyps, progressive weakness, lethargy, joint pain, anemia	extensive, crescentic, and necrotizing glomerulonephritis in 27 of 29 (93%) glomeruli, consistent with ANCA-mediated glomerulonephritis on light microscopy; on immunofluorescence, mesangial staining for IgA was observed, which met the criteria IgAN	high dose intravenous MTPD, plasmapheresis, and hemodialysis, after biopsy IV rituximab and oral prednisone, after 1 month CYC	minimal improvement in renal function → scheduled hemodialysis → kidney transplantation after 4 months	2021	(24)
19 y.o., female	anti-PR3 positive	irritation, redness, eye pain	IgAN	AZT, MTX	improvement	2010	(25)
32 y.o., male	anti-MPO negative, anti-PR3 positive	tracheal inflammation with histologic evidence of necrotizing vasculitis	mesangial hyperplasia	steroids, CYC	renal remission, pulmonary lesions persisted	1999	(26)
42 y.o., male	anti-PR3 positive	hematuria, proteinuria, pulmonary vasculitis	florid crescents in 3 of 6 (50%) glomeruli	steroids, RTX 375 mg/m² once weekly for 4 weeks and plasma exchange (7 sessions), MMF as maintenance for 2 years	improvement	2023	(27)
13 y.o., female	anti-MPO negative, anti-PR3 positive	microscopic hematuria, proteinuria, ankle joint pain, purple palpable purpura, hoarseness	Oxford score MEST-C: M_1_, E_0_, S_1_, T_0_, C_1_	MTPD 1 g/day for 3 days, prednisolone (1 mg/kg/day), and CYC (2 mg/kg/day)	improvement	2013	(28)
12 y.o., female	anti-PR3 positive	proteinuria, hematuria, anemia, keratitis, headache, morning stiffness, palpable purpuric lesion, pain of hip, thighs, arms, shoulders	focal crescentic and sclerosing glomerulonephritis ANCA associated with pauci-immune vasculitis	AZT (100 mg/day), prednisolone (27.5 mg/day)	improvement	2020	(29)

MEST-C criteria include mesangial hypercellularity (M0 and M1 corresponding to 50% and >50% of the glomeruli showing hypercellularity, respectively), endocapillary hypercellularity (E0: absent or E1: present), segmental glomerulosclerosis (S0: absent or S1: present), tubular atrophy/interstitial fibrosis (Tx, T0, T1, and T2 corresponding to unknown, 25%, 26–50%, and >50% of cortical area involvement, respectively), cellular/fibrocellular crescents (C0, C1, and C2 corresponding to their absence, presence in ≥1 and <25% of glomeruli, and presence in ≥25% of glomeruli, respectively) (30). IV, intravenous; PD, prednisone; MTX, methotrexate; AZT, azathioprine; CYC, cyclophosphamide; MTPD, methylprednisolone; MMF, mycophenolate mofetil; RTX, rituximab; anti-PR3, anti-proteinase-3; anti-MPO, anti-myeloperoxidase.

Consequently, one can infer that infections could be triggers of AAV and IgAN development. Rathman et al. assessed 270 patients with AAV to determine whether there is an association with prior respiratory tract infection. Surprisingly, the authors found that those infections are positively associated with MPO-ANCA but not PR3-ANCA vasculitis (34). Apart from NETs formation, infectious diseases could predispose to AAV via molecular mimicry, superantigens, and proinflammatory signaling. Infectious factors reduce immune functions and may cause persistent or recurrent autoimmunity in individuals who are genetically predisposed and/or in the context of environmental exposure (35).

Chebotareva et al. were the first to describe an effective therapy for ANCA-positive IgAN with rituximab (RTX) (17). A 19-year-old patient presented with AAV glomerulonephritis exacerbation 1 and 7 years after diagnosis. During the second exacerbation, a kidney biopsy revealed IgAN and the patient was administered RTX. Four weekly infusions of 500 mg RTX led to the remission of glomerulonephritis, which was maintained with mycophenolate mofetil (MMF).

Rao et al. first reported a patient with AAV and IgAN linked to silica exposure (18). The authors suggested that exposure to silica caused immune dysregulation that resulted in IgAN and AAV development. It is recommended to consider silica exposure as a putative trigger for autoimmune diseases.

Drugs may also trigger AAV and IgAN (2, 36). Galante et al. described the coexistence of AAV and IgAN triggered by propylthiouracil (19). According to the authors’ hypothesis, propylthiouracil-induced AAV resulted in an infiltration of IgG4-rich cells in the tubulointerstitial tissue as well as epiphenomena of anti-GBM antibody production. It is unknown whether AAV occurred first or was a result of mesangial IgA deposition.

Another study showed that, in comparison to ANCA-negative IgAN patients, ANCA-positive IgAN patients present a more severe clinical and histological image. Surprisingly, the renal prognosis was worse in ANCA-negative crescentic IgAN patients than in ANCA-positive individuals after aggressive short-term immunosuppressive therapy (37). Nonetheless, due to the relatively small sample size (20 patients), the treatment outcomes should be corroborated by future studies.

Although we were able to find several eligible reports on IgAN and AAV in the current literature, we do believe that coexistence could be more prevalent and current knowledge is limited. We hypothesize that underestimation could be a result of the paucity of research on IgAN and AAV due to researchers’ low priority for case reports, publishing policies that focus on original articles, economic issues, and underdiagnosis of IgAN. An IgAN diagnosis is made on the basis of a biopsy examination followed by immunofluorescence or immunohistochemical staining; such a procedure is not ordinarily performed.

## 4 Conclusions

Our patient with AAV presented symptoms from multiple organ systems. As regards renal manifestation, biopsy-proven IgAN preceded AAV glomerulonephritis diagnosis. Differences in the histological picture between the two performed biopsies were remarkable. CRP and other traditional inflammatory markers were elevated only during exacerbations. Thus, these were not applicable to monitor disease activity.

Emerging evidence demonstrates that overlapping AAV with IgAN results in a more severe clinical picture, rapidly decreasing kidney function, and various extrarenal manifestations. Thus, clinicians should acknowledge the possibility of ANCA-positivity in rapidly progressing IgAN. Whether there is causality between AAV and IgAN remains unknown. Future studies should be conducted to evaluate the putative common pathophysiology of these diseases.

Since the immunosuppressive protocol utilized in AAV was successful in patients with AAV and IgAN, ANCA testing may be beneficial for patients who have rapidly progressing IgAN for therapeutic and predictive purposes. As regards the management of IgAN associated with AAV, immunosuppressive treatment with methylprednisolone and cyclophosphamide is recommended. Alternatively, methylprednisolone with rituximab, plasma exchange, mycophenolate mofetil, and intravenous immunoglobulin (IVIG) could also be considered.

## Data availability statement

The original contributions presented in the study are included in the article/supplementary material. Further inquiries can be directed to the corresponding author.

## Ethics statement

Written informed consent was obtained from the individual(s) for the publication of any potentially identifiable images or data included in this article.

## Author contributions

Conceptualization: MT and MKu. Investigation: MT and MB. Resources: MT, PD, and MKu. Data curation: MT and PD. Writing—original draft preparation: MT, PD, MB, and MKu. Writing—review and editing: MT, PD, MKu, and MKr. Visualization: MT and PD. Supervision: MKu, PD, and MKr. Funding acquisition: MKr. All authors contributed to manuscript revision and read and approved the submitted version.
